# Sustaining China’s leadership in metabolic research: NSFC funding growth, equity challenges, and policy imperatives

**DOI:** 10.1093/lifemeta/loag027

**Published:** 2026-09-09

**Authors:** Shuo Li

**Affiliations:** Department of Life Sciences, National Natural Science Foundation of China, Beijing 100085, China

Over the past few decades, China has emerged as a global leader in scientific and technological innovation, driven by a strategic emphasis on fundamental research. A pivotal factor underpinning this progress is the substantial increase in research funding, enabled by the nation’s sustained economic growth and the rapid expansion of its scientific workforce. The Chinese government has systematically prioritized research and development (R&D), consistently elevating its investment as a percentage of gross domestic product (GDP). According to recent statistics, China’s R&D expenditure now accounts for nearly 2.5% of its GDP (as of 2022), with a growing proportion allocated to foundational scientific inquiries.

Metabolic research has become a cornerstone of China’s basic research agenda, particularly given the high prevalence of metabolic disorders such as diabetes, obesity, liver diseases, and cancer within the population. These conditions are intimately linked to dysregulation of metabolic pathways, necessitating mechanistic investigations to identify therapeutic targets. Chinese researchers have made seminal contributions to this field, including elucidating molecular mechanisms of metabolic dysfunction, discovering novel biomarkers, and pioneering translational interventions. Such advancements underscore the critical role of sustained governmental funding in enabling cutting-edge scientific discovery.

The National Natural Science Foundation of China (NSFC), a primary funding agency for scientific research in China, has been instrumental in advancing metabolic research through its diversified funding mechanisms. As illustrated in Fig. 1a, NSFC allocations to metabolic research have increased steadily over the past two decades, paralleled by a proportional rise in the number of funded projects (Fig. 1b). Funding distribution across programs remains stratified, with the General Program receiving the largest share, followed by the Young Scientist Program (Fig. 1c). All quantitative data presented in Figs. 1 and 2 were derived from the NSFC completed-project database. These investments have provided substantial support for China’s recent breakthroughs in metabolic science. Importantly, several dedicated funding mechanisms have been established to support metabolism-focused research. These include the NSFC Major Research Plan “Spatiotemporal Network Regulation of Glucose and Lipid Metabolism,” which addresses metabolite sensing, organelle crosstalk, inter-organ communication, metabolic reprogramming, and metabolic disease; and the NSFC Excellent Research Group Project (formerly the Basic Science Center Project) “Energy Metabolism and Health,” established in 2020 to investigate the regulation of energy metabolism across the life course and its links to metabolic diseases. Since 2024, the NSFC has also served as the lead agency for the National Key R&D Program “Developmental Programming and Its Metabolic Regulation.” Together, these initiatives reflect a shift from support for individual metabolic projects toward coordinated programs spanning molecular mechanisms, organ crosstalk, whole-body physiology, development, and disease.

**Figure 1:**
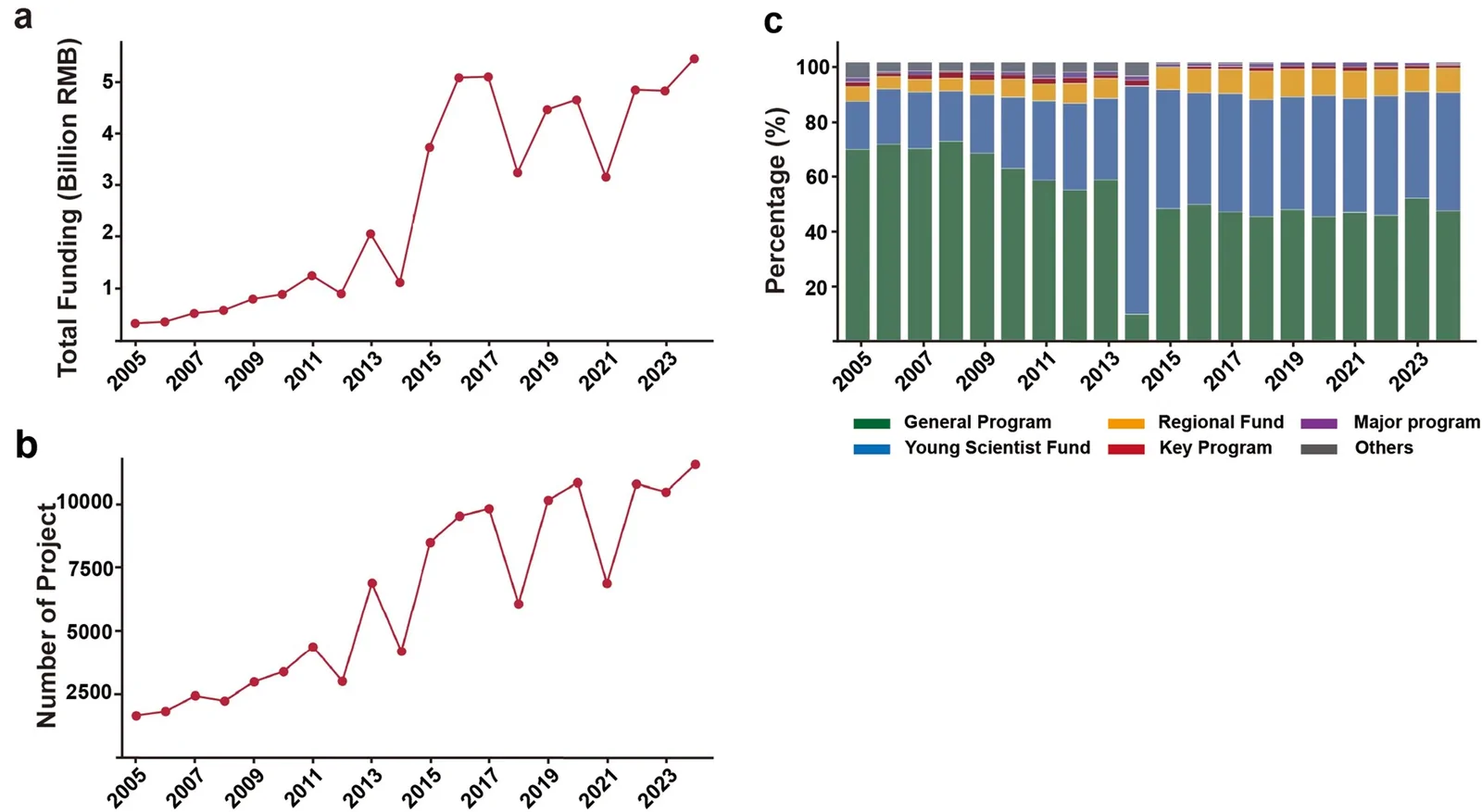
Funding trends for NSFC-supported metabolic research in China from 2005 to 2024. (a and b) Annual expenditure (a) and the annual number of funded projects (b) for metabolic research in China from 2005 to 2024. (c) Percentage of total recorded award amounts allocated to each program category within each completion cohort. All quantitative data were derived from the NSFC completed-project database.

**Figure 2:**
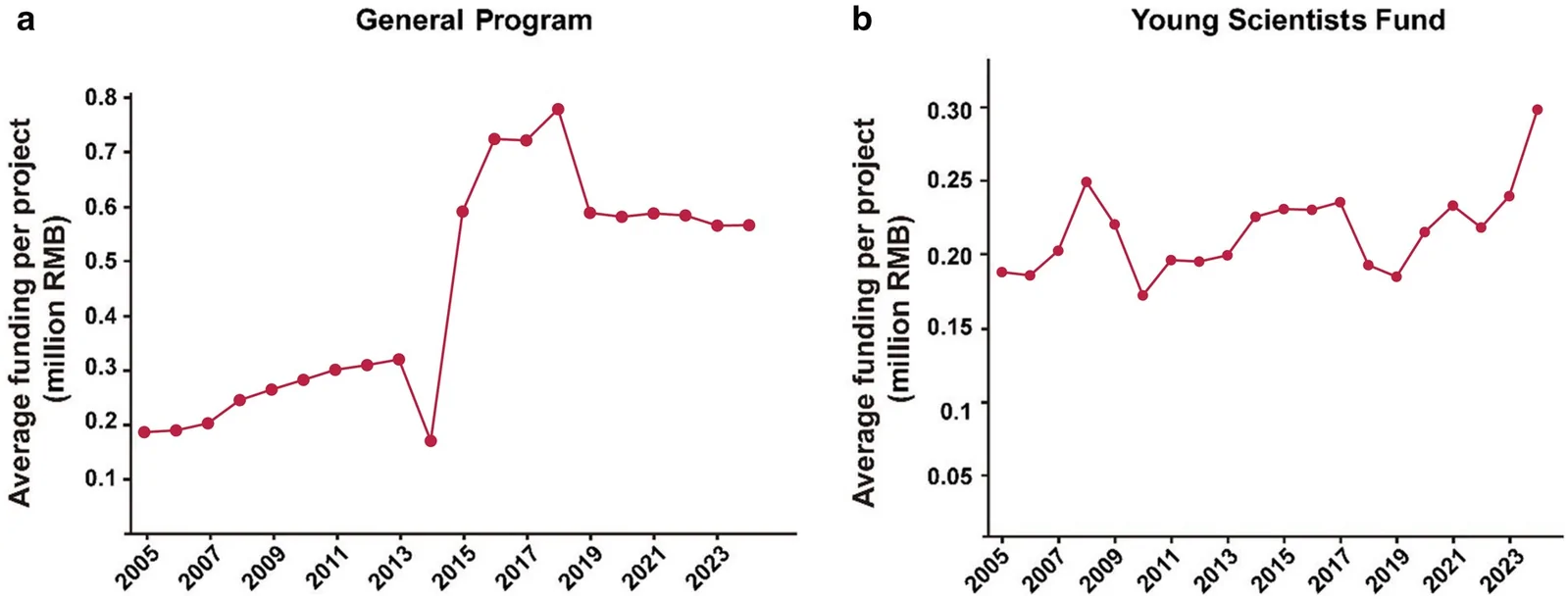
Average project budget for NSFC-supported metabolic research across various programs. (a and b) Average project budget for metabolic research in China from 2005 to 2024 for the General Program (a) and the Young Scientist Program (b). All quantitative data were derived from the NSFC completed-project database.

Despite this expansion in funding, critical challenges persist. Mean recorded funding per General Program project remained relatively stable across recent completion cohorts (Fig. 2a). For the Young Scientists Fund, the apparent increase between the 2023 and 2024 completion cohorts primarily reflected a change in the funding-accounting basis rather than an increase in the standard award (Fig. 2b). For projects completed before 2024, the recorded amounts generally represented direct costs. Beginning in 2021, the Young Scientists Fund adopted a lump-sum funding system, under which the recorded amount represents total funding. Consequently, for projects completed in 2024 and thereafter, the standard award is recorded as RMB 300 000, comprising RMB 240 000 in direct costs and RMB 60 000 in indirect costs (Fig. 2a and b). This disparity is particularly relevant for early-career investigators in metabolic research. Unlike many traditional studies, metabolic research increasingly requires integrated approaches spanning molecular mechanisms, cellular regulation, organ interactions, and whole-body physiology. Such studies often combine genetically engineered animal models, nutritional interventions, metabolic phenotyping, stable-isotope tracing, metabolomics, proteomics, and single-cell and spatial approaches. In addition, metabolic phenotypes are highly influenced by physiological context, including feeding status, circadian timing, age, sex, microbiota, and inter-organ communication, increasing the need for rigorous experimental design, adequate biological replication, and multilevel validation. Against this background, whether current per-project funding levels remain aligned with the increasing technical complexity and cost structure of modern metabolic research warrants further consideration. Current funding may constrain experimental scope, often forcing researchers to compromise on technological innovation (e.g., omitting advanced multi-omics approaches) or scale (e.g., limiting preclinical validation). Furthermore, intensifying competition has reduced success rates for early-career investigators, exacerbating career instability. Additional downstream consequences include delayed project timelines due to budget-driven protocol revisions, restricted access to state-of-the-art instrumentation, and challenges in recruiting specialized technical staff. It is important to note that fluctuations in project completions in certain years are due to adjustments in project duration (e.g., the General Programs had a duration of 3 years in 2010, which was extended to 4 years in 2011, resulting in fewer project completions in 2014). Importantly, these challenges have also prompted ongoing reforms within the funding system. The NSFC has emphasized directing limited scientific and technological resources toward frontline researchers with the greatest innovative potential. In line with this principle, the NSFC has continued to strengthen support for early-career researchers by expanding the scale of the Young Scientists Fund, establishing basic research programs for doctoral students, and providing longer-term and more stable support for outstanding early-career investigators. These efforts reflect a transition from simply expanding the research workforce toward more targeted support for emerging investigators and original research.

To sustain China’s leadership in metabolic research, strategic interventions are imperative:

- Increase and balance public and private investment to support the growing research workforce. Internationally harmonized Organization for Economic Co‑operation and Development (OECD) statistics show that businesses performed 77.7% of R&D in China and 78.4% in the United States in 2023. However, R&D performance should not be conflated with funding sources. In the United States, businesses performed 77% and funded 75% of total R&D in 2024, whereas the federal government remained the largest funder of basic research, accounting for 39.9% [1, 2]. These data indicate complementary roles: public funding is indispensable for long-term, high-risk basic research, while private investment is concentrated more heavily in applied research, experimental development, and commercialization. Therefore, augmenting overall investment from both public and private sectors to match the growing scientific workforce, with targeted fiscal and regulatory incentives to stimulate private-sector R&D and partnerships, is essential.
- Optimize allocation while safeguarding early-career scientists. International experience suggests that competitive funding systems should maintain a balance between supporting high-impact research and providing dedicated opportunities for early-career investigators. In the United States, the National Institutes of Health (NIH) Early Stage Investigator (ESI) policy provides additional consideration for early-career researchers during peer review, and evaluation of this ­policy showed that it helped stabilize access to major research funding for newly emerging investigators [3]. In Europe, the European Research Council (ERC) has established dedicated schemes including Starting Grants and Consolidator Grants to support researchers transitioning toward independence, while maintaining Advanced Grants to support established investigators pursuing frontier research. Similarly, the UK Research and Innovation (UKRI) Future Leaders Fellowship provides long-term support for outstanding early-career researchers to develop independent research programs. In China, further restructuring of allocation policies could prio­ritize high-impact projects while safeguarding early-career scientists, using tiered funding and collaborative grants to optimize resources and reduce fragmentation.
- Refine the current evaluation system. The NSFC has conti­nuously promoted reforms in evaluation practices, emphasizing the scientific significance and originality of proposed research rather than simple publication counts. Ongoing classified-review reforms within the NSFC and recent simplification of NIH peer review provide useful examples of how existing systems can evolve without discarding their core strengths.
- Strengthen standardized and responsible data sharing. Open data are particularly valuable in modern metabolic research because metabolomics, proteomics, imaging, single-cell, and spatial datasets are costly to generate but can support independent validation and secondary analysis. One survey found that only 18.3% of eligible biomedical studies discussed publicly available data and only 5.2% reported independent replication [4]. These findings highlight the importance of establishing more standardized and responsible data-sharing practices for publicly funded research. Therefore, mandatory open data policies for publicly funded outcomes should be implemented—within 12 months of publication, original data, code, tools, and protocols must be deposited in national repositories, with tiered access for sensitive data; sharing performance will be integrated into project evaluation and future funding priorities, supported by a dedicated data-governance fund to foster an ecosystem of data sharing, reuse, and discovery.
- Strengthen research integrity and build a sustainable research workforce. As research funding and scientific output continue to expand, maintaining a high-quality research ecosystem requires stronger safeguards for research rigor, reproducibility, data transparency, and responsible reporting. The NSFC has implemented a series of initiatives to promote research integrity, including strengthening oversight mechanisms through the Supervision Committee, improving procedures for investigating and handling research misconduct, publicly disclosing confirmed cases, and incorporating integrity requirements into research-funding management. Future efforts should further integrate research integrity into project application, review, and evaluation processes by considering reproducibility, data transparency, standardized data management, and responsible research practices. In parallel, workforce sustainability should be promoted through stable support mechanisms, cross-stage funding pathways, and improved career development opportunities for researchers at different career stages. Given the substantial burden of metabolic disease and the mechanistic breadth of contemporary metabolism research, a large and multidisciplinary research community remains scientifically justified. However, continued expansion should not be treated as an end in itself. Policy should shift from maximizing researcher numbers toward aligning workforce size and composition with stable funding, shared research infrastructure, rigorous training, reproducible output, and diverse career pathways. Regular assessment of scientific quality, unmet research needs, career outcomes, and available resources should guide future workforce growth.

In conclusion, sustaining China’s leadership in metabolic research will require not only continued investment but also a more balanced and resilient funding ecosystem. By aligning funding levels with research complexity, protecting early-career investigators, strengthening evaluation, data sharing, and research integrity, and building a sustainable workforce, China can better translate its expanding scientific capacity into durable, high-quality discoveries and long-term public-health benefits.
